# New Staphylinidae (Coleoptera) records with new collection data from New Brunswick, Canada: Oxyporinae

**DOI:** 10.3897/zookeys.186.2502

**Published:** 2012-04-26

**Authors:** Reginald P. Webster, Ian DeMerchant

**Affiliations:** 1 Natural Resources Canada, Canadian Forest Service - Atlantic Forestry Centre, 1350 Regent St., P.O. Box 4000, Fredericton, NB, Canada E3B 5P7

**Keywords:** Staphylinidae, Oxyporinae, new records, Canada, New Brunswick

## Abstract

Five species of Oxyporinae: *Oxyporus occipitalis* Fauvel, *Oxyporus quinquemaculatus* LeConte, *Oxyporus major* Gravenhorst, *Oxyporus rufipennis* LeConte, and *Oxyporus stygicus* Say, are newly recorded from New Brunswick, bringing the number of Oxyporinae known from the province to eight. The first documented records from New Brunswick are provided for *Oxyporus kiteleyi* reported by Majka et al. (2011). *Oxyporus occipitalis* and *Oxyporus major* are newly reported for the Maritime provinces of Canada. Collection and habitat data are presented for all these species.

## Introduction

This paper treats new Staphylinidae records from New Brunswick of the subfamily Oxyporinae. The Oxyporinae of the New World were reviewed by Campbell (1969, 1978). This Subfamily includes only the genus *Oxyporus* in North America. The biology and larva have been described for a number of the North American species (McCabe and Teale 1982; Leschen and Allen 1988; Hanley and Goodrich 1993, 1994; Goodrich and Hanley 1995b). Members of this genus exhibit an obligate association with mature Agaricales (gilled), Boletales (bolete), and Polyporales (polypore) mushrooms, and both larvae and adults feed on the spore-producing layer of the mushrooms (Hanley and Goodrich 1995b). The host preferences and behavior of the New World *Oxyporus* species were reviewed by Hanley and Goodrich (1995b). Members of this genus vary in the range of fungal host genera they use. For example, adults of *Oxyporus quinquemaculatus* LeConte have a narrow host preference range (*Pluteus* species), whereas other species, such as *Oxyporus vittatus* Gravenhorst, use a broad range of host genera of fungi, although the larvae of all species appear to have a narrower range of host species than the adults and are usually found in only one or two host fungi (Hanley and Goodrich 1995a, b). The short duration of the life cycle of only 14–17 days is probably an adaptation related to the ephemeral nature of the host fungi (Hanley and Goodrich 1993, 1994, 1995b; Goodrich and Hanley 1995).

Campbell and Davies (1991) reported eight species of *Oxyporus* for Canada and two species (*Oxyporus lateralis* Gravenhorst and *Oxyporus vittatus*) from New Brunswick. Majka et al. (2011) reported *Oxyporus kiteleyi* Campbell from New Brunswick but did not provide any supporting references or data. Here, five species are added to the faunal list of New Brunswick, and the first documented records from New Brunswick of *Oxyporus kiteleyi*, bringing the number of Oxyporinae known from the province to eight.

## Methods and conventions

The following records are based in part on specimens collected as part of a general survey by the first author to document the Coleoptera fauna of New Brunswick.

## Collection methods

Oxyporinaewere collected from mushrooms. Mushrooms were placed in a plastic box, broken into pieces, and the adults aspirated into a vial. A description of the habitat was recorded for all collections. Locality and habitat data are presented exactly as on labels for each record. This information, as well as additional collecting notes, is summarized in the collection and habitat data section for each species.

### Specimen preparation

A few examples of male specimens were dissected to confirm their identity. The genital structures were dehydrated in absolute alcohol and mounted in Canada balsam on celluloid microslides, and pinned with the specimens from which they originated.

### Distribution

Distribution maps, created using ArcMap and ArcGIS, are presented for each species in New Brunswick. Every species is cited with current published distribution in Canada and Alaska, using abbreviations for the state, provinces, and territories. New provincial records are indicated in bold under Distribution in Canada and Alaska. The following abbreviations are used in the text:

Acronyms of collections examined and referred to in this study are as follows:

**Table:** 

**AK**	Alaska	**MB**	Manitoba
**YT**	Yukon Territory	**ON**	Ontario
**NT**	Northwest Territories	**QC**	Quebec
**NU**	Nunavut	**NB**	New Brunswick
**BC**	British Columbia	**PE**	Prince Edward Island
**AB**	Alberta	**NS**	Nova Scotia
**SK**	Saskatchewan	**NF & LB**	Newfoundland and Labrador

**AFC** Atlantic Forestry Centre, Natural Resources Canada, Canadian Forest Service, Fredericton, New Brunswick, Canada

**CNC** Canadian National Collection of Insects, Arachnids and Nematodes, Agriculture and Agri-Food Canada, Ottawa, Ontario, Canada

**NBM** New Brunswick Museum, Saint John, New Brunswick, Canada

**RWC** Reginald P. Webster Collection, Charters Settlement, New Brunswick, Canada

## Results

Five species of Oxyporinae are newly recorded from New Brunswick, and the first documented records from New Brunswick of *Oxyporus kiteleyi*, bringing the number of Oxyporinae known from the province to eight (Table 1).

**Table 1.** Species of Oxyporinae (Staphylinidae) recorded from New Brunswick, Canada.

**Family Staphylinidae Latreille**
**Subfamily Oxyporinae Fleming**
*Oxyporus (Pseudoxyporus) lateralis* Gravenhorst
*Oxyporus (Pseudoxyporus) occipitalis* Fauvel**
*Oxyporus (Pseudoxyporus) quinquemaculatus* LeConte*
*Oxyporus (Oxyporus) kiteleyi* Campbell
*Oxyporus (Oxyporus) major* Gravenhorst**
*Oxyporus (Oxyporus) rufipennis* LeConte*
*Oxyporus (Oxyporus) stygicus* Say*
*Oxyporus (Oxyporus) vittatus* Gravenhorst

**Notes.** *New to province, **New to Maritime provinces.

## Species accounts

All records below are species newly recorded for New Brunswick, Canada, unless noted otherwise (additional records). Species followed by ** are newly recorded from the Maritime provinces (New Brunswick, Nova Scotia, Prince Edward Island) of Canada.

The classification of the Oxyporinae follows Bouchard et al. (2011).

### Family Staphylinidae, Latreille, 1802

**Subfamily Oxyporinae, Fleming, 1821**

#### Oxyporus (Pseudoxyporus) occipitalis

Fauvel, 1864**

http://species-id.net/wiki/Oxyporus_occipitalis

Map 1

##### Material examined.

**New Brunswick, Carleton Co.**, Meduxnekeag River Valley Nature Preserve, 46.1907°N, 67.6740°W, 23.VI.2006, R. P. Webster, mixed forest, in gilled mushroom (2 ♂, 5 ♀, RWC); Meduxnekeag River Valley Nature Preserve, 46.1940°N, 67.6800°W, 3.VII.2006, R. P. Webster, mixed forest, in gilled mushroom (1 ♂, 3 ♀, RWC).

**Map 1.**
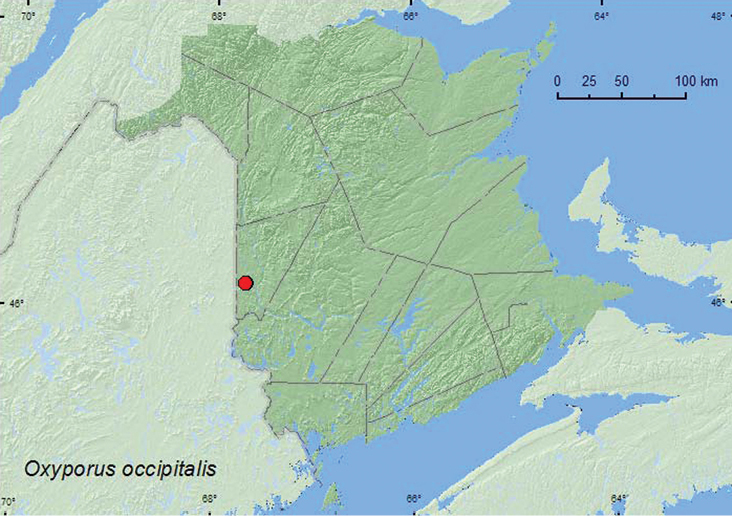
Collection localities in New Brunswick, Canada of *Oxyporus occipitalis*.

##### Collection and habitat data.

The biology, life history, and fungal hosts of *Oxyporus occipitalis* were reported by Hanley and Goodrich (1993, 1995a, b). This specieswasreported from 11 genera in seven families of fungi, but most individuals were reported from four genera (Hanley and Goodrich 1993, 1995a, b). In New Brunswick, adults were collected from various species (species not determined) of gilled mushrooms in mixed forests during June and July.

##### Distribution in Canada and Alaska.

YT,BC, AB, SK, MB, ON, QC, **NB** (Campbell 1969).

#### Oxyporus (Pseudoxyporus) quinquemaculatus

LeConte, 1863

http://species-id.net/wiki/Oxyporus_quinquemaculatus

Map 2

##### Material examined.

**New Brunswick, Albert Co.**, Caledonia Gorge P.N.A., (Protected Natural Area) 45.8257°N, 64.7791°W, 6.VII.2011, R. P. Webster, old hardwood forest (sugar maple and beech), on *Polyporus varius* (1, RWC). **Carleton Co.**, Meduxnekeag River Valley Nature Preserve, 46.1907°N, 67.6740°W, 23.VI.2006, R. P. Webster, mixed forest, in gilled mushroom (1 ♀, RWC); Meduxnekeag River Valley Nature Preserve, 46.1897°N, 67.6710°W, 25.VI.2007, R. P. Webster, mixed forest, in gilled mushroom (1 ♂, RWC); Meduxnekeag River Valley Nature Preserve, 46.1898°N, 67.6766°W, 2.VI.2008, R. P. Webster, mixed forest, in small brown gilled mushrooms on side of rotten log (3 ♂, RWC). **York Co.**, Charters Settlement, 45.8286°N, 66.7365°W, 11.VII.2006, 2.VI.2007, R. P. Webster, mature mixed forest, in gilled mushrooms (2 ♂, 1 ♀, RWC).

**Map 2.**
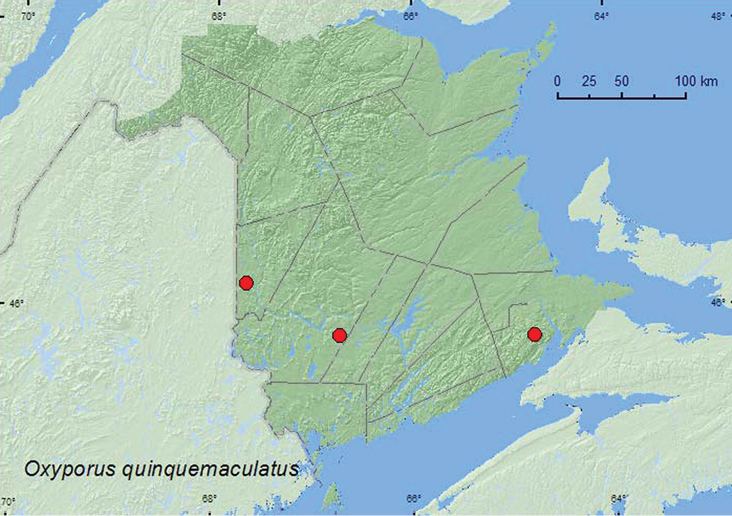
Collection localities in New Brunswick, Canada of *Oxyporus quinquemaculatus*.

##### Collection and habitat data.

*Oxyporus quinquemaculatus* has a relatively narrow range of hosts (five genera in three families), with most records from the genus *Pluteus* (Hanley and Goodrich 1995b). This species was also reported from *Laccaria amethystina* Murr., *Psilocybe spadicea* Fries, and *Naematoloma sublateritium* Karst. by Weiss and West (1920, 1921). In New Brunswick, this species was collected from gilled mushrooms (species not determined) and from *Polyporus varius* Fr. in mixed forests during June and July.

##### Distribution in Canada and Alaska.

ON, QC, **NB**, NS (Campbell 1969).

#### Oxyporus (Oxyporus) kiteleyi

Campbell, 1978

http://species-id.net/wiki/Oxyporus_kiteleyi

Map 3

##### Material examined. 

**Additional New Brunswick records, Carleton Co**., Meduxnekeag River Valley Nature Preserve, 46.1907°N, 67.6740°W, 19.VIII.2004, 8.VIII.2006, R. P. Webster, mixed forest, in *Boletus* sp. mushrooms (2 ♂, 1 ♀, RWC); Meduxnekeag River Valley Nature Preserve, 46.1896°N, 67.6700°W, 26.IX.2007, R. P. Webster, hardwood forest, on group of *Pholiota* sp. mushrooms at base of dead standing beech (1 ♀, RWC); Meduxnekeag River Valley Nature Preserve, 46.1878°N, 67.6705°W, 18.VIII.2008, R. P. Webster, hardwood forest,in large orange gilled mushrooms [probably *Gymnopilus spectabilis*] near base of dead standing beech tree (5 ♂, 6 ♀, RWC, NBM); same locality and collector, 2.IX.2008, hardwood forest,on large orange gilled mushroom [probably *Gymnopilus spectabilis*] on side of rotten beech log (2 ♂, RWC); Jackson Falls, Bell Forest, 46.2200°N, 67.7231°W, 7.VIII.2009, R. P. Webster, mature hardwood forest, on large orange gilled mushroom [probably *Gymnopilus spectabilis*] on side of rotten beech log (7, NBM, RWC).

**Map 3.**
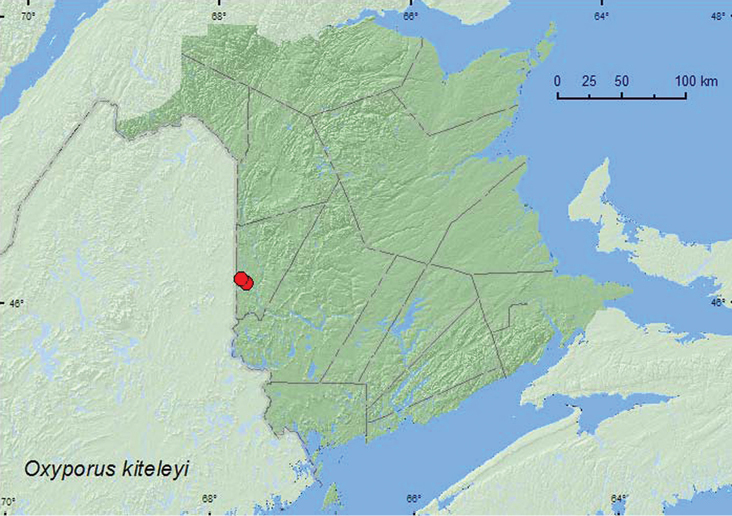
Collection localities in New Brunswick, Canada of *Oxyporus kiteleyi*.

##### Collection and habitat data.

Hanley and Goodrich (1995b)considered *Oxyporus kitelyi* to have a relatively narrow range of host species. Adults have been reported from *Suillus* sp. (Boletaceae) from Massachusetts and Georgia (Campbell 1978) and *Armillaria mellea* (Tricholomataceae) (Hanley and Goodrich 1995b). In New Brunswick, adults were found on *Boletus* sp. mushrooms (Boletaceae), *Pholiota* sp. (Cortinariaceae) at the base of standing dead American beech (*Fagus grandifolia* Ehrh.), and inside a large orange-gilled mushroom species (probably *Gymnopilus spectabilis* (Cortinariacae)) near bases of dead standing American beech trees or on rotten beech logs. Adults occurred in tunnels within the caps of the orange-gilled mushroom species. This species was collected during August and September.

##### Distribution in Canada and Alaska.

QC, NB (Campbell 1978). *Oxyporus kiteleyi* was listed as occurring in New Brunswick by Majka et al. (2011) without any supporting references or data. Here, we provide the first documented records from New Brunswick.

#### Oxyporus (Oxyporus) major

Gravenhorst, 1806**

http://species-id.net/wiki/Oxyporus_major

Map 4

##### Material examined.

**New Brunswick, Carleton Co**., Meduxnekeag River Valley Nature Preserve, 46.1907°N, 67.6740°W, 19.VIII.2004, 7.IX.2004, 14.IX.2005, R. P. Webster, mixed forest, in *Boletus* sp. mushrooms (3 ♂, 2 ♀, RWC).

**Map 4.**
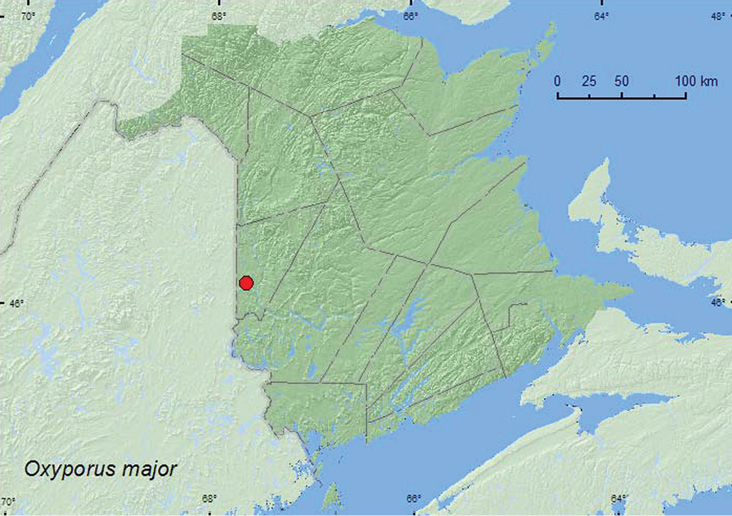
Collection localities in New Brunswick, Canada of *Oxyporus major*.

##### Collection and habitat data.

Campbell (1969) reported this species from a *Lactarius* sp. (Russulaceae). The biology, development, and a description of the larva of *Oxyporus major* were reported by Goodrich and Hanley (1995b). They reported this species from six families of fungi. Adults were most frequently collected from *Stropaharia hardii* Atkinson (Strophariaceae), *Lepiota acutaesquamosa* (Weinm.) Kummer (Lepiotaceae), and *Armillaria* spp. (Tricholomataceae). The only known larval host is *Stropaharia hardii* and *Lepiota acutaesquamosa* (Goodrich and Hanley 1995a, b). In New Brunswick, *Oxyporus major* was collected from *Boletus* sp. (Boletaceae) mushrooms during July, August, and September.

##### Distribution in Canada and Alaska.

QC, **NB** (Chagnon 1917). Campbell (1969) considered a record from Montreal, Quebec based on specimens in the Fauvel Collection as doubtful unless verified by additional collecting and, therefore, did not report this species from Canada. However, there was a record supported by a specimen from Quebec (Montreal Island) reported by Chagnon (1917) that confirmed the presence of this species for the province of Quebec and Canada. There are also recent specimens from Quebec in the R. Martineau Collection at the Laurentian Forestry Centre’s Insectarium in Quebec City, Quebec and in the CNC.

#### Oxyporus (Oxyporus) rufipennis

LeConte, 1863

http://species-id.net/wiki/Oxyporus_rufipennis

Map 5

##### Material examined.

**New Brunswick, Albert Co.**, Caledonia Gorge P.N.A., 45.7692°N, 64.8093°W, 12.IX.2011, R. P. Webster, old hardwood forest (sugar maple and yellow birch), on *Pholiota* sp. mushrooms on yellow birch log (1, NBM). **Carleton Co**., Meduxnekeag River Valley Nature Preserve, 46.1940°N, 67.6800°W, 23.VI.2006, 3.VII.2006, R. P. Webster, mixed forest, on *Pleurotus* sp. on dead standing trembling aspen (2 ♂, NBM, RWC). **Restigouche Co.**, Mount Carleton Prov. Park, Mount Bailey, 47.4042°N, 66.9189°W, 3.IX.2006, R. P. Webster, old hardwood forest, on mass of *Pholiota* sp. mushrooms on large dead standing yellow birch (5 ♂, 4 ♀ (over 50 individuals observed), RWC).

**Map 5.**
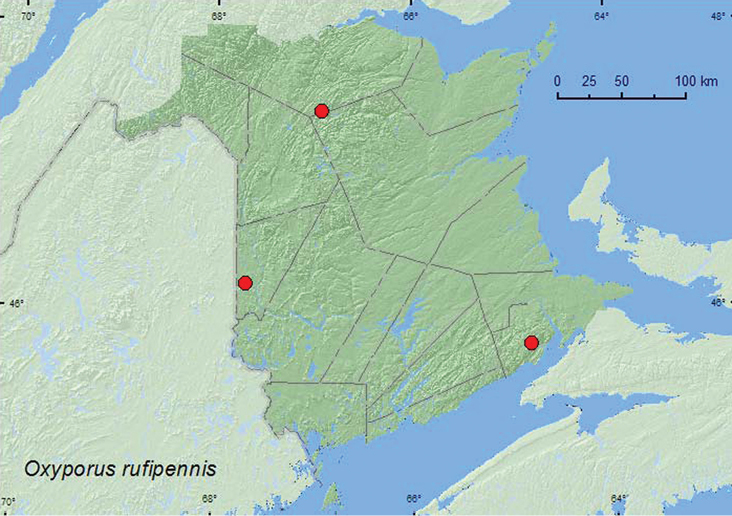
Collection localities in New Brunswick, Canada of *Oxyporus rufipennis*.

##### Collection and habitat data.

Hanley and Goodrich (1995b) considered *Oxyporus rufipennis* to have a relatively narrow range of host species (*Pholiota* (Cortinariaceae), *Polyporus* (Polyoraceae), *Omphalotus*, *Pleurotus* (Tricholomataceae)). In New Brunswick, this species was collected from mushrooms on standing trees and a recently fallen tree: *Pleurotus* sp. mushrooms on dead standing trembling aspen (*Populus tremuloides* Michx.), from masses of *Pholiota* sp. mushrooms on a large standing (partially dead) yellow birch (*Betula alleghaniensis* Britt.), and a recently fallen yellow birch. Adults were captured during June, July, and September.

##### Distribution in Canada and Alaska.

ON, QC, **NB**, NS (Campbell 1969; Campbell and Davies 1991).

#### Oxyporus (Oxyporus) stygicus

Say, 1831

http://species-id.net/wiki/Oxyporus_stygicus

Map 6

##### Material examined.

**New Brunswick, Carleton Co**., Meduxnekeag River Valley Nature Preserve, 46.1940°N, 67.6800°W, 23.VI.2006, R. P. Webster, mixed forest, in *Boletus* sp. mushrooms (2 ♂, 1 ♀, RWC); Becaguimec Island in Saint John River, 46.3106°N, 67.5392°W, 13.IX.2006, R. P. Webster, mature mixed forest, on *Pholiota* sp. mushrooms on log (1 ♂, 3 ♀, NBM, RWC). **Sunbury Co.**, Lakeville Corner, 45.9007°N, 66.2423°W, 10.IX.2006, R. P. Webster, silver maple forest, on *Boletus* sp. mushroom (2 ♂, RWC).

**Map 6.**
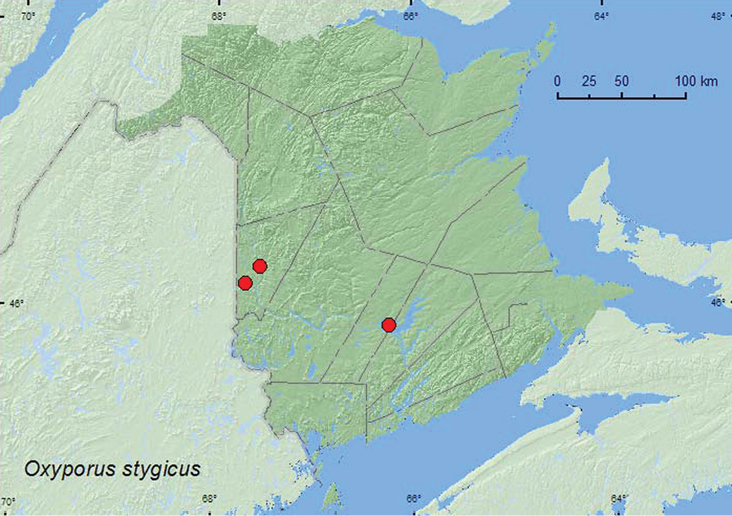
Collection localities in New Brunswick, Canada of *Oxyporus stygicus*.

##### Collection and habitat data.

The biology, development, and a description of the larval characteristics of *Oxyporus stygicus* were reported by Hanley and Goodrich (1994). They reported this species from three families of fungi: Cortinariaceae (*Pholiota*), Polyporaceae (*Grifola*, *Polyporus*), and Tricholomataceae (*Armillaria*, *Omphalotus*, *Pleurotus*). Large series of immatures were collected from *Pholiota aurivella* (Fr.) Kummer, *Pholiota* sp., and *Omphalotus illudens* (Schw.) Bigelow. Weiss and West (1920) reported *Oxyporus stygicus* from *Pleurotus ostriatus* Fries. Hanley and Goodrich (1995b) considered*Oxyporus stygicus* to have a relatively narrow range of host species compared with other *Oxyporus* sp. This species was collected from *Boletus* and *Pholiota* spp. mushrooms in mixed forests and a silver maple (*Acer saccharum* Marsh) forest in New Brunswick. Adults were collected during June and September.

##### Distribution in Canada and Alaska.

QC, **NB**, NS (Campbell 1969).

## Supplementary Material

XML Treatment for Oxyporus (Pseudoxyporus) occipitalis

XML Treatment for Oxyporus (Pseudoxyporus) quinquemaculatus

XML Treatment for Oxyporus (Oxyporus) kiteleyi

XML Treatment for Oxyporus (Oxyporus) major

XML Treatment for Oxyporus (Oxyporus) rufipennis

XML Treatment for Oxyporus (Oxyporus) stygicus
